# Decisional needs among patients and physicians in the treatment of chronic myeloid leukaemia: a qualitative analysis in the Netherlands

**DOI:** 10.1136/bmjopen-2025-112705

**Published:** 2026-01-22

**Authors:** Simone Mingels, Manon J J Cloots, Yolba Smit, Nicole M A Blijlevens, Eduard F M Posthuma, Andre L A J Dekker, Rianne R R Fijten, Esri Wener, Corien C G Kromkamp

**Affiliations:** 1 Research Institute for Oncology and Reproduction GROW, Maastricht University, Maastricht, Netherlands; 2 Department of Radiation Oncology MAASTRO, Maastricht University Medical Centre+, Maastricht, Netherlands; 3 Institute for Nutrition and Translational Research in Metabolism NUTRIM, Maastricht University, Maastricht, Netherlands; 4 Department of Gastroenterology and Hepatology, Maastricht Universitair Medisch Centrum+, Maastricht, Netherlands; 5 Department of Hematology, Radboud universitair medisch centrum, Nijmegen, Netherlands; 6 Department of Internal Medicine, Reinier de Graaf Groep, Delft, Netherlands; 7 Department of Hematology, Leiden University Medical Center, Leiden, Netherlands; 8 CMyLife Program, A.T. TWINTEY, Eindhoven, Netherlands; 9 Department of Oncology, Rijnstate Hospital, Arnhem, Netherlands

**Keywords:** HAEMATOLOGY, Decision Making, Leukaemia, Patient-Centered Care

## Abstract

**Abstract:**

**Objective:**

Treatment advancements in chronic myeloid leukaemia (CML) have made the disease manageable but carry significant risk of side effects. Bridging information gaps between patients and physicians through shared decision-making (SDM) is increasingly favoured, yet understanding treatment complexities remains a challenge. This study sought to identify decisional and informational needs of both patients and physicians in CML care.

**Design:**

A qualitative study using semi-structured interviews was conducted to investigate the opinions, attitudes and preferences of both patients with chronic myeloid leukaemia and physicians.

**Setting:**

Patients and physicians were recruited through the Dutch CMyLife platform, an initiative of haematologists, patients and patient organisations. They were provided with the participant information and invited to participate if interested.

**Participants:**

A total of 15 interviews (n=10 patients, n=5 physicians) were conducted between April and October 2023.

**Primary and secondary outcome measures:**

A pre-defined interview guide was developed based on the Decisional Needs Assessment questionnaire. Interview transcripts were thematically analysed.

**Results:**

Eight themes and 28 sub-themes were observed, highlighting patient needs, treatment choices and informational preferences. Patients emphasised the importance of understanding medication options and side effects, while physicians stressed the necessity of delivering up-to-date and comprehensible information. Almost all participants had experienced professionals making the treatment decision, without patient involvement, especially when initiating treatment. Some patients expressed too little information and missed partnership with professionals at treatment onset. Peer support, decision-making dynamics and the role of caregivers were also significant considerations.

**Conclusions:**

Both shared and distinct perspectives on CML treatment decision-making between patients and physicians were revealed, underscoring the complexity of decisional needs in CML management. The findings emphasise the importance of patient-centred care, SDM and tailored communication strategies to optimise patient outcomes and satisfaction. Improved communication and evidence-based decision-making tools can significantly impact patient well-being. Further research and interventions are necessary to address the challenges in decision-making processes in CML care.

**STRENGTHS AND LIMITATIONS OF THIS STUDY**

- The semi-structured open nature of the interviews allowed for a deep understanding of personal experience and expanding of knowledge on decisional needs.
- The recruitment of active patients through the CMyLife platform and the relatively small sample size may not have fully captured the experiences of all patients through the possible introduction of selection bias in the study.
- Inclusion of patients and physicians from multiple centres across the Netherlands may enhance the generalisability of findings to the broader population.

## Introduction

 Chronic myeloid leukaemia (CML) is a type of blood cancer characterised by the uncontrolled growth of myeloid cells in the bone marrow.^1^ This abnormal growth is caused by a genetic mutation that leads to the formation of a fusion gene, known as BCR::ABL1.^1 2^ To block the excessive growth and division of myeloid cells, treatment with tyrosine kinase inhibitors (TKIs) is standard care.^2^ TKIs have transformed CML from a life-threatening condition to a usually manageable chronic malignant disease with a life expectancy almost equivalent to the normal population, enabling patients to lead relatively normal lives with ongoing treatment and regular monitoring.^3^,^5^ However, these treatments carry significant risks of severe side effects, particularly cardiac adverse events.^6^ Monitoring and, if necessary, switching medication are crucial for patients with pre-existing cardiac conditions, predispositions towards cardiac disease or those experiencing severe side effects.^7^ Medication discontinuation may be necessary for those intolerant or resistant to TKI treatment.^8^ These decisions are generally initiated by the treating physician, guided by clinical guidelines and standard practice.^2 9 10^

Patients with CML often have different goals and concerns compared with their physicians, including a greater need for information and understanding about potential side effects.^11^ This highlights the need for improved communication and shared decision-making (SDM) practices in CML care. SDM empowers patients by granting them greater autonomy in their treatment decisions.^12^ It fosters patient engagement by providing them with comprehensive information to assess the benefits and risks of various treatment options, thereby enhancing their understanding of potential side effects and enabling their inclusion in the monitoring process.^12 13^ Navigating SDM in cancer care can be challenging due to the need to fully understand the benefits, harms and uncertainties of treatment.^14^ Additionally, certain systemic cultural constraints within clinical practice may hinder the effective implementation of SDM.^15^ The literature shows that patients increasingly prefer active involvement in healthcare decisions, though this varies depending on the specific decision and patient characteristics.^16^ To facilitate the process of SDM in the selection of the most suitable treatment for patients with CML, this study sought to identify decisional and informational needs of both patients and physicians in CML care.

## Methods

### Study design

A qualitative study with semi-structured interviews was used to investigate the opinions, attitudes and preferences of both patients with chronic myeloid leukaemia and physicians. This allowed for exploration of patterns and themes while still permitting comparisons between respondents. The Standards for Reporting Qualitative Research guidelines were used.^17^ A pre-defined interview guide based on the Decisional Needs Assessment questionnaire^18^ was used uniformly across all interviews, as it contains pre-defined broad questions that trigger conversation. Distinct versions were tailored for patients with chronic myeloid leukaemia to ascertain their decision-making needs and for healthcare practitioners to identify areas for enhancing patient support during decision-making (see onlinesupplemental files 1^2^). Each version included broad questions that triggered conversation regarding SDM.

### Researcher characteristics and reflexivity

SM was a first-year PhD candidate in the Clinical Data Science department at Maastricht University with 2 years’ experience in qualitative analysis; her research focuses on communication about AI and patient decision aids. MJJC was a third-year PhD candidate in a research group developing decision aids for inflammatory bowel disease. RRRF is an assistant professor in Clinical Data Science and supervisor of SM and MJJC. Her group focuses on AI development and implementation, patient decision aid development and SDM. None of the researchers had a treatment relationship with participants. We anticipated that our backgrounds in clinical data science, AI, decision-aid development and SDM could orient questioning and interpretation toward communication, information needs and decision support.

### Study participants

At the start of the study, participants were recruited using convenience sampling from two populations, as recruitment was facilitated through collaboration with a third-party intermediary. Initially, patients were enlisted through contact with the Dutch CMyLife network,^19^ an initiative of Radboud university medical centre in which haematologists, patients and patient organisations support and improve the quality of care for individuals with a haematological malignancy. Patients from different hospitals across the Netherlands were informed about the study via the CMyLife team and given participant information, consisting of a study rationale and consent form. If interested, patients could provide their contact details for further contact with our research team for participation in the study. Written informed consent was obtained before the interview. Patients were informed about the burden of the intervention and time required to participate in the research. Second, physicians were recruited by the CMyLife team, after which they were contacted by our research team, where they were provided with the participant information and invited to participate if interested. Patients were included in the study if they were of adult age (>18 years) and were previously diagnosed with CML. Physicians were included if they were actively practising medicine within a Dutch hospital and regularly treated patients with CML (at least monthly). Data saturation was concluded when no new emergent themes were detected.

### Data collection and analysis

Demographic information of participants was collected at the start of each interview. Interviews were conducted by two researchers (MJJC, RRRF), online via Microsoft Teams or over the phone, based on the digital literacy of the participant. Interviews lasted between 30 and 60 min. Video and/or audio recordings of interviews were transcribed and anonymised. Transcripts were coded using Atlas.ti software for qualitative data analysis (V.23). Two researchers (MJJC, SM) independently generated inductive codes for selections of the transcript relevant to the aim of this research, according to codebook thematic analysis.^20^ Themes were developed by comparing codes within and across transcripts, clustering related codes and examining patterns and relationships. Theme definitions were iteratively refined through discussions and comparisons. Any disagreements between coders were resolved through discussions with a third researcher (RRRF) to reach consensus. Any confusion regarding answers of participants was resolved by contacting said participant, asking for clarification. Participants were not asked to provide feedback on the results.

### Patient and public involvement

This research was initiated by the CML patient organisation, which raised the research question based on patient-identified needs. Patients and patient representatives were involved in shaping the research aims and contributed to the study design. Identifying topics related to patient preferences in CML care may provide a foundation for supporting patients during decision moments throughout their treatment journey and facilitate SDM. It may even form the basis of the content and format of a decision aid, which can be used to disseminate information to a wider CML community. Participants could opt to receive the study results, which will be shared after publication with those who expressed interest.

## Results

### General characteristics

A total of 15 interviews were conducted between April and October 2023, consisting of patients (n=10) and physicians (n=5). None of the patients or physicians contacted by the research team were excluded following initial recruitment. The education level is indicated according to the European Qualifications Framework.^21^ Decision moments that emerged during the interviews included the following: starting medication, switching between medication, changing medication dose, pausing medication and stopping medication. All patients experienced more than one decision in their CML disease trajectory, even those with a disease duration of 1 year. The demographic results are summarised in table 1.

**Table 1:** Summary participant characteristics

	Patients (n=10)	Physicians (n=5)
Sex		
M	5	2
F	5	3
Median age (range)	64 (56–71)	54 (31–60)
Education		
EQF2	1	
EQF4	3	
EQF6	6	
Median disease duration (range)	7 (1–19)	
Discipline		
Internist haematologist		4
Nurse practitioner		1
Decisions discussed		
Starting medication	9	
Switching between medication	7	
Changing medication dose	8	
Pause of medication	3	
Stopping medication	3	

A total of eight themes and 28 sub-themes related to treatment decisions and informational needs were identified (see table 2). Patients and physicians had a different mention of subthemes in their interviews (visualised in online supplemental file 3). The overall decisional needs and example quotes of both patients and physicians are summarised in table 3.

**Table 2:** Emergent themes and subthemes

Theme	Subtheme
TKI side effects	Physical side effects Psychological side effects
TKI choice	Guidelines, indications and preferences Starting medication Switching medication Changing medication dose Pause of medication Stopping medication Quality of life
Decision-making	Decision made by patient Decision made together with patient and physician Decision made by physician
Additional caregivers	Nurse practitioner General practitioner Psychologist Satisfied with treating physician
Informational characteristics	Comorbidities Online information Paper-based information Understandable information Prediction model and personalised information Information provision
Informational content	Medication options Understanding CML
Peer support	Online peer support Real-life peer support
Relatives	Involved relatives Role of relatives

CML, chronic myeloid leukaemia; TKI, tyrosine kinase inhibitor.

**Table 3:** Decisional needs and example quotes of patients and physicians

Theme	Patients	Physicians	Example quotes
Discussed treatments and side effects	Clear information regarding medication options, their requirements of use and side effects, predominantly focused on their effect on daily life and activities.	Clear information regarding medication options and their side effects, predominantly focused on severe physical side effects.	*“Oh yes, I’m a bit old school, so I usually make a choice for the patient. I explain that ‘I want this and I want to give you that, for this and this reason”. (physician E*)
Treatment choices	Information on the options in treatment continuation regarding switching of medication, adjusting of medication dose, discontinuation of treatment and the consequences of treatment non-adherence.	Consideration of clinical guidelines, medication pricing, medical indications, quality of life, autonomy and motivation and individual preferences and experiences with medications.	*“When I mentioned that my complaints remained such that it significantly affected my life and my daily activities, he offered me the option of taking another medication.” (patient D*)
	Effective dialogue between patients and physicians in decision-making processes.	Collaborative choice between physician and patient, or based on the physician’s recommendations.	*“That’s right, that’s the problem. The distance between doctor and patient is just too great. They have to be able to find each other.” (patient B*)
Professional caregivers	The possibility to discuss treatment options with the treating physician or nurse practitioner in an empathic manner.		*“I was then transferred from one hospital to another. And the first thing they ask is: ‘How are you actually doing? How do you feel?’. And I thought that was really brilliant.” (patient A*)
Informational characteristics	Reliable, accessible, comprehensible and consistent information, preferably through online resources, while guaranteeing privacy.	Current information, preferably through online resources, that is reliable and given by the physician.	*“And I had already read everything. The moment I knew I had CML, I immediately started looking.” (patient B*)
	Effective and understandable communication with their healthcare providers.	An effective manner of communicating information to patients.	*“Well, armed to the teeth, that’s how I always go to the visits with the haematologist. I do feel like I really, well almost, let’s say have to develop to her level in order for the conversations to run smoothly. It is, that’s a bit of a challenge.” (patient G*)
	The possibility of personalised information, with or without predictive models, based on individual preference.	The possibility of personalised information, with or without predictive models, while ensuring reliability.	*“No, I don’t want to know. No, that’s the risk, that’s the risk. I think that maybe unconsciously to live like that, I would just get scared.” (patient H*)
	More input and information at diagnosis regarding treatment selection.	Reassurance during initial phase and not overwhelming patients with disease-related information.	*“There was no choice, so you can talk about it, but doesn’t make much sense. If you want to survive, you just have to start taking those pills.” (patient I*)
Informational content	Comprehend the advantages and disadvantages associated with different treatment choices, based on individual preference.	Explaining various medication options to patients, including anticipated reactions and possible side effects.	*“You try to explain as best as possible what the side effects are, what the current guidelines are, and how much response you expect based on the profile the patient has.” (physician A*)
	Review information retrospectively.	For patients to understand the nature of the disease.	*“The first thing you hear is that you have leukaemia. And you’re like, will I still see my kids turn 18? That’s the first thing you think. Then you come home, and the initial shock is over, and then you want to ask for information. What is CML?” (patient C*)
Peer support	Forum/discussion group, both online as well as physical.	Inform patients about forums or peer support contacts, preferably patients with similar treatment expectancies or well-being.	*“My experience is that most people who want to participate in discussion groups do so because they have a certain, yes, those people often have slightly more negative experiences than positive ones.” (physician D*)
Relatives	The involvement of a family member or other significant person in treatment support.		*“Especially at the beginning of the treatment process, or if things are not going well, they usually bring someone with them. That is usually either the partner or a child.” (physician B*)

### Discussed treatments and side effects

Patients and physicians were asked which medication they received or previously prescribed, along with the conditions influencing these treatment choices. Both groups discussed TKIs such as dasatinib, imatinib and nilotinib. In contrast, asciminib, bosutinib and ponatinib were exclusively discussed by physicians, while one patient referred to tucatinib. Discussions surrounding medication options underscored the adverse effects associated with treatment (see online supplemental file 4). Certain aspects, such as the frequency of intake, were not always consistently recalled by patients, particularly with dasatinib. Some patients were not able to specify their medication while describing the experienced side effects. Patients tended to focus on the impact of treatment on daily life and activities, for example, muscle pain or cramps and fatigue/malaise. In contrast, physicians spoke more about strictly physical side effects of the treatments, such as gastrointestinal issues and fluid retention.

### Treatment choices

Patients were asked to reflect on what, if any, treatment choices they made during their disease duration, and which role they played in these decisions. Physicians were asked how they make decisions regarding treatment for the patients and what role patients may play in this. All physicians reported the use of guidelines in their decision-making, although patients did not always perceive this consistency. All physicians noted that their selection of medication was partially guided by their individual preferences and experiences with medications.

In agreement with the Dutch guidelines,^22^ most physicians preferred to initiate treatment with Imatinib, and in compliance, many patients started their treatment with Imatinib. Reasons for this preference included the reasonable safety and limited toxicity, compromising the often-experienced side effects by patients. Several medical indications were mentioned for deviating from the standard medication regimen, such as the desire to become pregnant, risk of cardiovascular problems, diabetes or a mutation. Physicians also indicated that the quality of life and autonomy and motivation of the patient influenced the medication choice. When patients experienced a significant number of side effects, many switched medication, while some adjusted their medication dose, took a break from their medication or discontinued the medication altogether. Additionally, some patients mentioned several other reasons for switching medication, such as cost-saving measures, mutation, lack of efficacy or elevation of BCR::ABL1. Dosage adjustments were also made based on favourable or unfavourable blood values or cost-saving measures. Discontinuation of medication could result from achieving a good response and disease control.

Physicians rarely indicated that a patient made their treatment choice independently, whereas patients more frequently reported having done so. A majority of the patients and physicians indicated that medication choices were made in collaboration or based on the physician’s recommendations. Patients repeatedly emphasised the importance of effective dialogue between themselves and their physicians in decision-making processes. Nearly all patients and all physicians reported that at least one of the treatment decisions was not a collaborative effort, but rather solely determined by the physician. This narrative was predominant with patients at the start of their disease trajectory. On several occasions, this occurred on the patient’s request, due to fear of making the decision or feeling unprepared to choose their medication. Physicians also noted some instances where patients themselves expressed a preference against making their own choice.

### Professional caregivers

Patients and physicians were asked about the role that professional caregivers may play to support both groups. Most patients expressed a need to discuss treatment options with their treating physician or nurse practitioner. Physicians acknowledged that nurse practitioners can aid in treatment decision-making, if empathetic and considerate. Only one patient felt the desire to discuss their disease and options with their general practitioner. Furthermore, one patient and one physician suggested that patients could benefit from guidance from a psychologist. Overall, patients were satisfied with their current attending physician.

### Informational characteristics

Patients and physicians were asked how disease-related information and information about treatment options can best be fabricated and disseminated, and which conditions they must meet. In addition, both groups were asked about their own experiences with this. Patients outlined several prerequisites that information must meet before dissemination to patients, namely reliability, accessibility and alignment among various informational sources. There was a call for vigilance regarding the privacy implications of certain informational sources, particularly of some online platforms. One physician emphasised the importance of keeping information up to date. Most physicians indicated directing patients to websites, in agreement with most patients who expressed a preference for digital information resources. Some patients also independently sought information online, primarily through patient platforms, although a physician cautioned that consulting online sources may not always be ideal due to the potential risk of encountering misinformation. Some patients expressed a preference for accessing their personal medical records through online hospital systems. Moreover, some patients were offered paper-based informational materials in the form of pamphlets by their physicians. Interestingly, an equal number of patients expressed both a desire and a lack of interest in receiving pamphlets.

Both groups highlighted the need for conveyed information to be comprehensible. All physicians acknowledged the challenge of effectively communicating information to patients. Patients indicated varying preferences as to whether they require training to facilitate communication with healthcare providers or not. Most patients emphasised a desire for personalised risk assessment, including the chance of success of treatment and side effects based on patient characteristics, while others had no interest. Some patients and physicians expressed interest in predictive models for patient care, highlighting the importance of reliability in such models.

Furthermore, there is a notable disparity regarding the timing of information provision to patients. Physicians advocated against overwhelming patients with disease-related information during the acute or initial phase post-diagnosis and underscored the significance of reassurance. Moreover, many patients expressed significant anxiety during the period surrounding the diagnosis of CML. Some patients, however, indicated that at the time of diagnosis they had too little engagement in the decision and received too little information.

### Informational content

Patients and physicians were asked which disease-related information they think is important to give or receive, and which information they already gave or received previously. All physicians stressed the importance of explaining the various medication options to patients. Most patients expressed a fundamental need to understand the advantages and disadvantages associated with different treatment choices and often expressed a desire to review such information retrospectively. Some physicians also underscore the importance of informing patients about the anticipated reactions and side effects that may occur. According to most physicians, it is also important for patients to understand the nature of the disease itself. However, most patients themselves did not express this need.

### Peer support

Patients and physicians were asked about their opinion of support of fellow patients in different settings. Many patients express a need for a discussion group to exchange experiences and interact with others who share similar experiences. Specifically, this includes a preference for online forums, as well as offline alternatives. Most physicians indicate that they inform patients about forums or peer support contacts, with the condition that accessibility remains a priority. However, they do not distinguish between online and in-person contact. Additionally, one physician noted that peer support and discussion groups may not always align with treatment expectations or patient well-being.

### Relatives

Patients and physicians were asked about the role that relatives play in the patient’s disease journey. Patients typically do not attend consultations with their physician alone. Patients themselves indicate bringing their partner to consultations and mention their involvement in the decision-making processes. Physicians also frequently observed the presence of partners during consultations, alongside (adult) children, parents and caregivers. While typically characterised as taking a supportive role, some individuals also offer advice, and in certain instances, they assume a leadership role.

## Discussion

The present study used qualitative methodology to investigate the decisional needs of patients and physicians in management of CML treatment. The findings revealed both commonalities and differences. Patients emphasised the impact of treatment on daily life, whereas physicians focused on physical side effects, although they acknowledged the importance of quality of life and patient autonomy. This reflects earlier work demonstrating a mismatch between patient-reported and physician-reported symptom burden in chronic haematological conditions such as CML, where physicians frequently underestimate the extent to which side effects affect patients’ quality of life.^23 24^ Therefore, enhanced communication and SDM are crucial to establish patient involvement in decision-making and adapt information preferences explicitly for each patient encounter.^11 25^

This study provides insights suggesting that while several medication decisions were subject to SDM, initial decisions were often physician-led. In some cases, this reflected patients’ preference to defer responsibility, but others recalled feeling excluded. Prior research has shown that these patients may view the decision-making process more negatively compared with those engaged in collaborative or autonomous decision-making.^13 26 27^

Patients frequently described seeking additional resources online motivated by the desire for more information on symptoms, prognosis and treatment options. In addition, they expressed their interest in forums or discussion groups with fellow patients with chronic myeloid leukaemia. Such support groups can offer emotional support and guidance from those sharing similar experiences.^28 29^ Physicians highlighted the importance of patients understanding their disease, while patients stressed the need to understand the advantages and disadvantages of various treatment options. Informed patients often feel more empowered in managing their disease and are more likely to adhere to treatment.^29^ Tailored information considering content, delivery and timing may address patients’ perceived information needs.^30 31^ However, effectively communicating risk information to the public presents a challenge, requiring careful consideration of potential risk overestimations.^32^

Physicians advocate against overwhelming patients with extensive disease-related information during the acute or initial phase post diagnosis,^11^ as excessive information can be confusing and overwhelming, especially in the initial stages of a patient’s journey.^29^ Patients, however, often felt they received insufficient information during this period. Research shows that patient satisfaction is higher when information needs are met early in the cancer journey.^33^ Many patients described the time of diagnosis to be stressful and anxious, emphasising the value of revisiting treatment information retrospectively, which is in line with earlier research.^34^ Remembering detailed medical information is challenging, particularly among older patients and those with poor prognosis or high anxiety levels.^35^,^37^ Effective communication between physician and patient has been shown to reduce anxiety and uncertainty, enhancing patients’ ability to recall prognosis and treatment options.^38^

This study provides valuable insights into the perspectives of patients with chronic myeloid leukaemia and physicians regarding decisional needs. However, several limitations should be considered. Not all potential medication options were discussed with patients, possibly due to adherence to medication guidelines by physicians limiting patient exposure to certain medications. In addition, the retrospective design may introduce recall bias among patients and physicians. However, this design allowed us to understand personal experience and expand our knowledge on decisional needs. Moreover, selection bias may be present since participants were recruited through the Dutch CMyLife platform, which may have led to the inclusion of predominantly active and involved patients in our study. Nonetheless, including patients and physicians from multiple centres across the Netherlands may enhance the generalisability of findings to the broader population.

In conclusion, our findings are in line with wider literature and illustrate the complexity of decisional needs in CML management. These highlight the similarities, but also the gap between patient and physician perspectives on symptoms, quality of life and information needs. Addressing these gaps requires a patient-centred approach, emphasising SDM and tailoring communication strategies to optimise patient outcomes and satisfaction. Future research should examine whether a well-designed decision aid with reliable information could improve patient-physician communication and empower patients with chronic myeloid leukaemia to have a more active role in the decision-making process.

## Supplementary material

10.1136/bmjopen-2025-112705 online supplemental file 1

10.1136/bmjopen-2025-112705 online supplemental file 2

10.1136/bmjopen-2025-112705 online supplemental file 3

10.1136/bmjopen-2025-112705 online supplemental file 4

## Data Availability

All data relevant to the study are included in the article or uploaded as supplementary information.
