# Supplementary material for: Decisional needs among patients and physicians in the treatment of chronic myeloid leukaemia: a qualitative analysis in the Netherlands
Source: BMJ Open. 2026 Jan 22;16(1):e112705. doi: 10.1136/bmjopen-2025-112705 (PMC12829388; doi:10.1136/bmjopen-2025-112705)
Supplement: online supplemental file 1 [file bmjopen-16-1-s001.pdf]

Interview guide for patients

Good morning/afternoon/evening. My name is ____________ of ____________ and I am conducting a questionnaire to learn more about people's decision-making needs when making choices regarding chronic myeloid leukaemia (CML). This information will help us to develop better educational materials for people who are faced with these choices. All information we collect in this voluntary questionnaire will be kept confidential. We would like your help, it takes about 60 minutes to complete. I'm going to give you some examples of health choices that some people with CML face. For example, some people have to make the following choice about which medication to take in order to start CML treatment or switch medications.

Decision

1. At this point, what do you think are the most important choices that people with CML face?
2. Let's focus on a specific choice. The choice to **(depending on patient):**

a. Switching medications due to side effects or response.
b. Medication to start with.
c. Stopping medication.

<table>
<tr>
<td>3. Let's talk about the difficulty of making this choice about CML. How do you feel or felt when you had to make this choice?

____
____
____
____
____
____
____
____
____________________</td>
<td>[Examine Behavioral Expressions of Decision Conflicts]

Do you feel/felt:
● Unsure of what to do
● Worried about what could go wrong
● Saddened or upset
● Constantly thinking about the choice
● Hesitating between choices or changing your mind
● Postponing the choice
● Unsure what's important
● Feeling physically stressed, tense muscles, rapid heart rate, difficulty sleeping</td>
</tr>
</table>

________________________________	
4. What makes the choice difficult for you?<br><br>________________________________<br>________________________________<br>________________________________<br>________________________________<br>________________________________<br>________________________________<br>________________________________<br>________________________________<br>________________________________<br>________________________________<br>________________________________<br>________________________________<br>________________________________<br>________________________________	*[Examine Factors Contributing to Decision Conflicts]*<br><br>**Are/do you feel:**<br>● Lack of information about options, benefits and distress<br>● Lack of information on the likelihood of benefits and distress<br>● Confused by too much information<br>● Confused by lack of clarity about what's important<br>● does not support themselves in making choices<br>● pressure from others<br>● Lack of motivation or not ready to make a choice<br>● Not enough skill to make a choice

5. Thinking about this choice, what were the options you had? (suggestions: dose, type of medication)
6. What do you see as the main advantages and disadvantages/risks of the options?

Option	Advantages	Disadvantages/Risks
1.		
2.		

3.		

<table>
<tr>
<td>7. Who else might be involved in making this choice with you?

____
____
____
____________________</td>
<td>[Examine Role in Decisive Behavior]

Do they usually:
Making the choice for you
Sharing the choice with you
Offering support or advice to you to make your own choice</td>
</tr>
</table>

<table>
<tr>
<td>8. How did you make this choice? And how would you have liked to make this choice?

____
____
____
____
____
____
____
____________________</td>
<td>[Investigate Decision Behavior]

You:
Gets information about options
Gets information about the opportunities for benefits and risks
Considers the personal interests of the benefits and risks
Gets information about how others make such a choice
Gets support from others
Finds ways to deal with the pressure</td>
</tr>
</table>

9. What would help you make this choice?
10. What would hinder you in making this choice?
11. Is there anything else that would help you overcome the impediment in making choices?
12. I would like to mention a number of options that can help you make a choice. Which of these would you like to use?

● Guidance from another healthcare provider	If so, please specify which types (e.g. GP, nurse practitioner)
● Discussion groups made up of people facing the same choice	If so, specify what type of organization or group
● Information material	If yes, please specify content<br>● Health Status<br>● Options<br>● Advantages<br>● Risks<br>● Potential for benefits/risks<br>● Help with Weighing Benefits vs. Risks<br>● Guidance in the steps of consultation and communication<br>● Other, namely ________________
	If yes, specify formatting<br>● Booklet/pamphlet<br>● Internet<br>● Videos/DVDs<br>● Other, namely ________________

13. Is there anything else that would help you better in supporting you in making your choice?

Characteristics of the patient

14. Age Range (Estimate)

- Twenty-something
- Thirty-something
- Forty-something
- Fifty-something
- People in their sixties or older

15. Gender (observe)

- Male
- Female

16. What is the highest level of education you have completed?

- Primary education
- Secondary education (vmbo, havo, vwo)
- Secondary vocational education (MBO)
- Higher professional education (HBO)
- Academic education (WO)

17. Duration of experience with the health problem ________________________________

[THANK THE RESPONDENT]
