# Supplementary material for: Decisional needs among patients and physicians in the treatment of chronic myeloid leukaemia: a qualitative analysis in the Netherlands
Source: BMJ Open. 2026 Jan 22;16(1):e112705. doi: 10.1136/bmjopen-2025-112705 (PMC12829388; doi:10.1136/bmjopen-2025-112705)
Supplement: online supplemental file 2 [file bmjopen-16-1-s002.pdf]

Interview guide for physicians

Good morning/afternoon/evening. My name is ____________ of ____________ and I conduct interviews with practitioners and patients to learn more about people's decision-making needs when making choices regarding chronic myeloid leukaemia (CML). During the interview, we will ask you some questions about the health choices that people may make in your field, for example selecting the appropriate treatment for CML. This information will contribute to a better understanding of patient decision-making needs to improve the planning of support in the choice. All the information we gather in this interview is kept confidential. We would like your help, it takes about 60 minutes to complete.

Decision

1. What choices do patients with CML have to make in your practice?
2. Let's focus on a specific choice. The choice on which medication to choose to treat their disease:

3. Let's talk about the difficulty people have in making this choice about CML treatment. How do patients feel when making this choice?	*[Examine Behavioral Expressions of Decision Conflicts]*<br><br>**Do patients feel:**<br><br>● Unsure of what to do<br>● Worried about what could go wrong<br>● Saddened or upset<br>● Constantly thinking about the choice<br>● Hesitating between choices or changing your mind<br>● Delaying the choice<br>● Unsure what's important to them<br>● Feeling physically stressed, tense muscles, rapid heart rate, difficulty sleeping
4. What makes the choice difficult for patients?	*[Examine Factors Contributing to Decision Conflicts]*

______________________________<br>______________________________<br>______________________________<br>______________________________<br>______________________________<br>______________________________<br>______________________________<br>______________________________<br>______________________________<br>______________________________<br>______________________________<br>______________________________<br>______________________________<br>______________________________<br>______________________________<br>______________________________	**Patients:**<br>● Lack of information about options, benefits and distress<br>● Lack of information on the likelihood of benefits and distress<br>● Are confused by too much information<br>● Perceived lack of clarity about what is important to them<br>● Feeling unsupported in making choices<br>● Feeling pressure from others<br>● Lack motivation or don't feel ready to make a choice<br>● Lack of ability or skill to make a choice

5. What do you see as the main options available to patients?
6. What do you see as the main advantages and disadvantages/risks of the options?

Option	Advantages	Disadvantages/Risks
1.		
2.		

3.		

7. What is your usual role in making this choice?<br><br>______________________________<br>______________________________<br>______________________________<br>______________________________	*[Research Role]*<br><br>**Do you usually:**<br><br>● Making the choice for the patients<br>● Sharing the choice with the patients<br>● Providing support or advice to patients to make their own choices

8. What factors make it difficult for you to support your patients in making the choice?

9. What factors make it easier for you to support your patients in making the choice?

10. Besides yourself and the patient, who else is usually involved in making this choice?<br><br>______________________________<br>______________________________<br>______________________________	*[Research]*<br><br>● Husband<br>● Family<br>● Friend<br>● Provider<br>● Other, namely ________________

11. What is their usual role in making this choice (i.e. the person mentioned above)	*[Research Role]*<br><br>**Do they usually:**<br><br>● Making the choice for the patients

______________________________<br>______________________________<br>______________________________<br>______________________________<br>______________________________	● Sharing the choice with the patients<br>● Providing support or advice to patients to make their own choices<br>● I don't know<br>● Other, namely _______________

12. How does the patient usually make such a choice?<br><br>______________________________<br>______________________________<br>______________________________<br>______________________________<br>______________________________<br>______________________________<br>______________________________<br>______________________________<br>______________________________	*[Research Behavior Choice]*<br><br>**They:**<br><br>● Get information about options<br>● get information about the opportunities for benefits and risks<br>● Consider the personal interests of the benefits and risks<br>● Get information about how others make such a choice<br>● Get support from others<br>● Finding ways to deal with pressure

13. What would help patients make this choice?
14. What would hinder patients from making this choice?
15. Is there anything else that would help to overcome the obstacle in making choices?
16. I list a number of possible ways to help some people with a choice, which ones do you think might be useful for your patients?

● Guidance from another healthcare provider	If so, please specify which types
● Discussion groups with people who are facing the same choice	If so, specify what type of organization or group
● Information material	If yes, please specify content

	• Health Status<br>• Options<br>• Advantages<br>• Risks<br>• Potential for benefits/risks<br>• Help with Weighing Benefits vs. Risks<br>• Guidance in the steps of consultation and communication<br>• Other, namely _______________
	If yes, specify formatting<br>• Booklet/pamphlet<br>• Internet<br>• Videos/DVDs<br>• Other, namely _______________

17. Is there anything else that would help you do a better job of supporting your patient's choice?

Characteristics of the practitioner

18. Age Range (Estimate)

- Twenty-something
- Thirty-something
- Forty-something
- Fifty-something
- People in their sixties or older

19. Gender (observe)

- Male
- Female

20. Discipline practiced, specify ______________________________________

21. Specialty practiced, specify ____________________________________

22. Practice location, specify ____

[THANK THE RESPONDENT]
