# Supplementary figures and images for: Decisional needs among patients and physicians in the treatment of chronic myeloid leukaemia: a qualitative analysis in the Netherlands
Source: BMJ Open. 2026 Jan 22;16(1):e112705. doi: 10.1136/bmjopen-2025-112705 (PMC12829388; doi:10.1136/bmjopen-2025-112705)

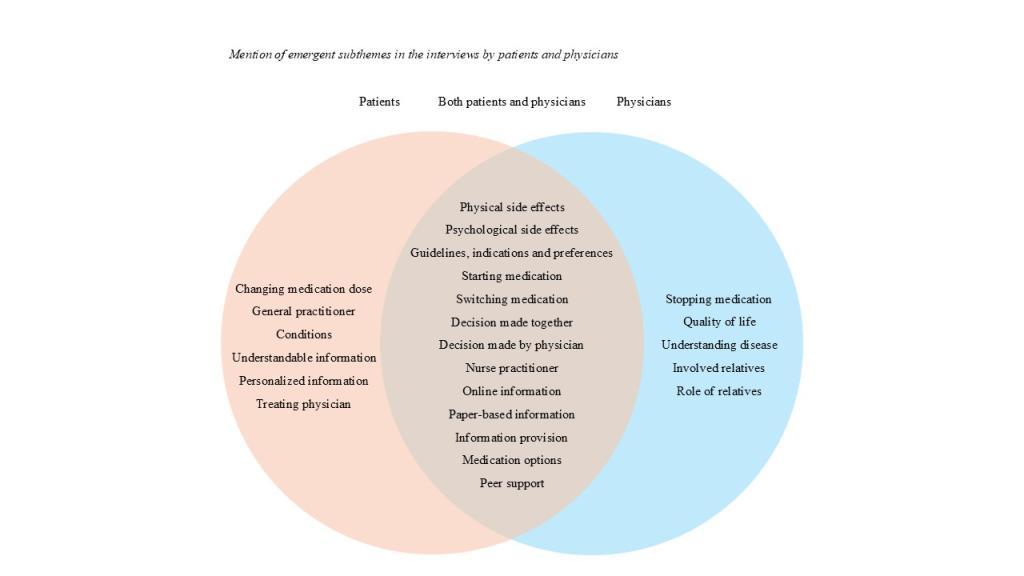

Supplement: online supplemental file 3 [file bmjopen-16-1-s003.jpeg]
