# Supplementary material for: Decisional needs among patients and physicians in the treatment of chronic myeloid leukaemia: a qualitative analysis in the Netherlands
Source: BMJ Open. 2026 Jan 22;16(1):e112705. doi: 10.1136/bmjopen-2025-112705 (PMC12829388; doi:10.1136/bmjopen-2025-112705)
Supplement: online supplemental file 4 [file bmjopen-16-1-s004.pdf]

Discussed medication options, side effects, and characteristics			
**Medication**	**Side effects and characteristics**	**Number of patients mentioned** (%)	**Number of physicians mentioned** (%)
Imatinib	Muscle pain/cramps	4 (40)	1 (20)
	Bone pain	2 (20)	0 (0)
	Joint pain	1 (10)	0 (0)
	Fatigue/malaise	3 (30)	1 (20)
	Gastrointestinal issues	1 (10)	4 (80)
	Fluid retention	0 (0)	2 (40)
	Swollen eyes	0 (0)	1 (20)
	Cardiovascular risk	1 (10)	0 (0)
	Diabetic risk	1 (10)	0 (0)
	Dietary restrictions	1 (10)	0 (0)
Nilotinib	Itchiness	0 (0)	1 (20)
	Hair loss	0 (0)	1 (20)
	Gastrointestinal issues	1 (10)	1 (20)
	Fluid retention	0 (0)	1 (20)
	Fatigue/malaise	0 (0)	1 (20)
	Cardiovascular risk	2 (20)	4 (80)
	Intake moment	1 (10)	3 (60)
	Taking on empty stomach	2 (20)	3 (60)
Dasatinib	Pleural effusion	3 (30)	2 (40)
	Pulmonary hypertension	0 (0)	1 (20)
	Taking on empty stomach	1 (10)	0 (0)
	Several intake moments	3 (30)	2 (40)
	Fatigue/malaise	1 (10)	0 (0)
Tucatinib	Taking on empty stomach	1 (10)	0 (0)
	Several intake moments	1 (10)	0 (0)
Asciminib	Taking on empty stomach	0 (0)	1 (20)
	Intake moment	0 (0)	1 (20)
Ponatinib	Cardiovascular risk	0 (0)	1 (20)
Bosutinib	Diarrhoea	0 (0)	3 (60)
	Nausea	0 (0)	1 (20)
	Skin rashes	0 (0)	1 (20)
	Liver test abnormalities	0 (0)	1 (20)
