# Supplementary material for: Decisional needs among patients and physicians in the treatment of chronic myeloid leukaemia: a qualitative analysis in the Netherlands
Source: BMJ Open. 2026 Jan 22;16(1):e112705. doi: 10.1136/bmjopen-2025-112705 (PMC12829388; doi:10.1136/bmjopen-2025-112705)
Supplement: Supplementary file 5 [file bmjopen-2025-112705.reviewer_comments.pdf]

PEER REVIEW HISTORY

BMJ Open publishes all reviews undertaken for accepted manuscripts. Reviewers are asked to complete a checklist review form and are provided with free text boxes to elaborate on their assessment. These free text comments are reproduced below.

ARTICLE DETAILS

Title (Provisional)

Decisional needs among patients and physicians in the treatment of Chronic Myeloid Leukaemia: A qualitative analysis in the Netherlands

Authors

Mingels, Simone; Cloots, Manon J.J.; Smit, Yolba; Blijlevens, Nicole M.A.; Posthuma, Eduard F.M.; Dekker, Andre L.A.J.; Fijten, Rianne R.R.; Wener, Esri; Kromkamp, Corien C.G.

VERSION 1 - REVIEW

**Reviewer**	**1**
**Name**	**Richardson, Daniel**
**Affiliation**	**UNC**
**Date**	**12-Dec-2024**
**COI**	**None**

This study sought to identify decisional and informational needs of both patients and physicians in CML care.
N=10 pts, n=5 physicians
The authors identified 5 themes related to decisional and informational needs. This is an important area of study as CML care is becoming increasingly preference sensitive. Overall, the manuscript lacked clarity and depth of synthesis and interpretation. Themes were too vague to be helpful (e.g. “personal experiences”) and the discussion lacked a deep understanding of prior work and implications to be highly valuable. While the manuscript offers some insightful thoughts, it could be substantially improved with a more thoughtful analysis of the data and generation of themes. Overall, I was left still with wanting a more deep dive into how patients weighed treatment decisions and their experiences in SDM for consequential decisions.
Particular comments/suggestions follow:

Theme 1 – I'm unsure whether the theme title "Treatment options" is appropriate as it seems that this fits more with side effects. I don't know the distinction between treatment options and treatment choices in the next theme. A deeper dive into how options were presented to patients from physicians, or how patients chose between options would have been helpful.
In general, it would be helpful in the top line of the paragraph describing each theme to succinctly summarize the theme presented rather than jump into some of the findings.
Pg 10 – line 6 – Unclear what is meant by the discussion of individual TKIs. Perhaps the distinction is between patients and physicians?
Treatment choices – In discussing imatinib as first line, are there Dutch recommendations/guidelines for this? That would be helpful to guide readers as guidelines change country by country.
"Child wish" is unclear; does this refer to desiring to become pregnant?
Additional caregivers theme is unclear. Does this mean additional clinician support, additional referrals? Typically, caregivers (in American English) refer to family members who provide care for patients. I'm unsure what a nurse specialist refers to. The example quote does not seem to fit.
Informational characteristics – What information is being referred to, and what is meant by "before dissemination"? This theme is very broad, discussing preference for reliable information about the disease to needing training for communication with physicians and to desire for personalized risk assessment. There is also information about lack of involvement in the decision-making process.
Informational content – What informational content is being referred to? Content in SDM with physicians? In flyers, websites, etc?
The theme of personal experiences is also too broad to be helpful.
It appears that one of the takeaways is that patients frequently felt left out of treatment decisions in the early stages and physicians noted frequently making choices for patients. This could be explored more in detail in the discussion. Several other studies have noted this, especially for adults with blood cancers. How should this be addressed? Are there ethical considerations? Paternalism v. patient autonomy discussion seems appropriate here.
Another conclusion that is reached is that tailored communication strategies are needed. Could you expand on the literature of decisional preferences in leukemia care or strategies to tailor communication to patients? Others have published on this topic including the Control Preference Scale, but I am unaware of studies that have looked specifically at CML.
The final comment about the development of a decision aid is important. Could you expand upon why a decision aid would be helpful? Most patients were satisfied with their care.
Given the small sample size, it is hard to justify the connected figure in how physicians and patients differ in their decisional needs.

VERSION 1 - REVIEW

**Reviewer**	**2**
**Name**	**Hewison, Ann**
**Affiliation**	**University of York, Department of Health**
**Sciences**	
**Date**	**12-Dec-2024**
**COI**	**None**

‘Yes Doctor, No Doctor’: A Qualitative Analysis of Decisional Needs Among Patients and Physicians in Chronic Myeloid Leukaemia Treatment
Thank you for the opportunity to review this interesting paper. The authors describe and discuss findings of patient and physician interviews regarding treatment decision making in chronic myeloid leukaemia (CML). It describes the characteristics of treatment decisions, perceptions of who makes decisions, and features of information and support that are valued. This may be useful to those developing interventions targeted at supporting shared decision making in CML where treatment options are increasing complex. However, the paper would benefit from a more clear and comprehensive description of the qualitative methods used and a more in depth analysis and presentation of the qualitative data. I have suggested changes (below), some of which I would consider major. I understand that word limit may have had an influence in some of these areas:
Study objectives/Abstract
• In the abstract: ‘Primary and secondary outcome measures’, states ‘Interview transcripts were thematically analysed through consecutive open, axial and selective coding.’ Open, axial and selective coding is usually associated with grounded theory methods rather than thematic analysis. A clearer description of the methods would be useful here (see related comments in Methods).
Methods
• Study design: This heading may be misleading as the text seems to describe data collection. It would read more sequentially if this section is renamed ‘data collection’ and is placed after the ‘patient and public involvement’ section.
• Study design: The method used is described as ‘a qualitative research method,’ this section would be strengthened by describing and referencing exactly what approach and methods were used and an explanation as to why they were chosen.
• Study design: A short statement justifying why the Decisional Needs Assessment questionnaire as a basis for the qualitative interview would be helpful and how this is

in line with your chosen qualitative methods/approach.
• Study design: When describing the interviewers, it would be helpful to know of any characteristics which may influence their data collection and/or interpretation (i.e. researcher reflexivity).
• Patient and public involvement: This heading is somewhat misleading as the text appears to describe the study sample and setting, not patient and public involvement. Re-naming the section ‘Study sample and setting’ would be clearer.
• Patient and public involvement: It would be interesting to know why convenience sampling was used and how many patients were excluded from the final sample.
• Data analysis: this section suggests that both thematic analysis and grounded theory approaches or methods were used. It would benefit from further explanation of how/if these two approaches/methods were integrated?
• Data analysis: this section reports that the study is of an ‘explorative nature’, and together with the reference to grounded theory above, suggests that the aim is to derive meaning from the data. However the aim and findings appear more focussed and descriptive. The section would benefit from a more comprehensive and consistent description of the data collection and analysis approach.
• Data analysis: It would strengthen this section if there was a little more description of the process of open, axial and selective coding and how these were built into the themes described in the results section.
Results
• General characteristics: It would be useful to explain the relevance of reporting the number of patients/physicians who mentioned each theme/subtheme (including table 2) , this seems a little unusual in a qualitative study which usually focusses on the meaning of themes rather than quantifying them.
• Themes: It would be interesting to know why numbers of participants relating to each theme/subtheme are reported, this seems a little unusual: see above comment
• Themes: themes are clearly described however there does not appear to be an obvious analysis of meaning within the data or the development of a theory, which does not seem entirely consistent with the use of grounded theory.
• Themes, table 4: this is a helpful summary. Some of the quotations provide context to, and suggest subtleties in, decision making which are not entirely reflected in the results section, such as the influence on decision making of patient-physician relationships and patients’ fears and anxieties. Further analysis of the qualitative data may result in a more in-depth understanding of decision making in CML.
Discussion
• This section confirms that several findings are supported by wider literature. It would be interesting and relevant to also relate findings to literature specific to CML and haematological malignancies regarding patient/physician decision making/consultation, such as the mismatch of patient and physician perspectives on symptom burden in CML and unmet information needs in patients with haematological malignancies. Additionally, relating the findings to other qualitative work in CML and/or haematological malignancies would be particularly relevant.

References
• This section would benefit from including more related literature related to CML and haematological malignancies, including qualitative studies.
• There is no reference for thematic analysis, which if used, would be required.

VERSION 1 - AUTHOR RESPONSE

Reviewer 1, Dr. Daniel Richardson, UNC. Comments to the Author:

- Theme 1 – I'm unsure whether the theme title "Treatment options" is appropriate as it seems that this fits more with side effects. I don't know the distinction between treatment options and treatment choices in the next theme. A deeper dive into how options were presented to patients from physicians, or how patients chose between options would have been helpful.
  Treatment options refer to the range of medication that patients have received and give us a clearer understanding which medication options are used in daily practice. It also helps us understand to which medications these side effects refer to. Treatment choices refer to the decision-making process of treatment of both patients and physicians, and how these choices are justified. I understand the confusion in this regard. Therefore, the fist theme has been changed to "discussed treatments & side effects".
- In general, it would be helpful in the top line of the paragraph describing each theme to succinctly summarize the theme presented rather than jump into some of the findings.
  Great suggestion, this has been added.
- Pg 10 – line 6 – Unclear what is meant by the discussion of individual TKIs. Perhaps the distinction is between patients and physicians?
- *Yes, it is added to showcase the difference in known and used TKIs between patients and physicians. It has been rephrased to make it more clear.*
- Treatment choices – In discussing imatinib as first line, are there Dutch recommendations/guidelines for this? That would be helpful to guide readers as guidelines change country by country.
  Yes, this agrees with the Dutch guidelines. A statement has been added.
- "Child wish" is unclear; does this refer to desiring to become pregnant?
  Yes, this refers to the wish of becoming pregnant. It has been changed.
- Additional caregivers theme is unclear. Does this mean additional clinician support, additional referrals? Typically, caregivers (in American English) refer to family members who provide care for patients. I'm unsure what a nurse specialist refers to. The example quote does not seem to fit.
  Nurse specialist also translates to nurse practitioner. The focus of this part is on additional professional caregivers. It has been rephrased. The quote refers to the added value of these caregivers, as they usually have more time to invest in the patient's wellbeing.

- Informational characteristics – What information is being referred to, and what is meant by “before dissemination”? This theme is very broad, discussing preference for reliable information about the disease to needing training for communication with physicians and to desire for personalized risk assessment. There is also information about lack of involvement in the decision-making process.
  Informational characteristics refer to how disease-related information about treatment options can best be fabricated and disseminated, and which conditions they must meet. I hope the added statement at the start makes this clearer. It is a broad theme that describes all prerequisites that should be considered before disease-related information is shared with patients.
- Informational content – What informational content is being referred to? Content in SDM with physicians? In flyers, websites, etc?
  Informational content refers to which subjects of information patients/physicians (wish to) receive/give. Hopefully the added statement at the start makes this clearer. This is independent of the informational characteristics.
- The theme of personal experiences is also too broad to be helpful.
  These subjects were important experiences of patients that did not fit any other theme. However, they should probably not be disregarded.
- It appears that one of the takeaways is that patients frequently felt left out of treatment decisions in the early stages and physicians noted frequently making choices for patients. This could be explored more in detail in the discussion. Several other studies have noted this, especially for adults with blood cancers. How should this be addressed? Are there ethical considerations? Paternalism v. patient autonomy discussion seems appropriate here.
  This is an important discussion that should be further investigated. However, due to word count restraints and scope, this is out of scope of this paper. This is a great topic that could be revisited in future research.
- Another conclusion that is reached is that tailored communication strategies are needed. Could you expand on the literature of decisional preferences in leukemia care or strategies to tailor communication to patients? Others have published on this topic including the Control Preference Scale, but I am unaware of studies that have looked specifically at CML.
  Idem.
- The final comment about the development of a decision aid is important. Could you expand upon why a decision aid would be helpful? Most patients were satisfied with their care.
  A lot can still be improved in the care that patients receive. Especially in the communication between patient and physician. Hence, the decision aid.
- Given the small sample size, it is hard to justify the connected figure in how physicians and patients differ in their decisional needs.
  This is something we noticed in this study. Validation in future research is necessary.

Reviewer 2, Dr. Ann Hewison, University of York. Comments to the Author:

- In the abstract: ‘Primary and secondary outcome measures’, states ‘Interview transcripts were thematically analysed through consecutive open, axial and selective coding.’ Open, axial and selective coding is usually associated with grounded theory methods rather than thematic analysis. A clearer description of the methods would be useful here (see related comments in Methods).
  This was wrongly described. The methods are in line with thematic analysis. It has been adjusted.
- Study design: This heading may be misleading as the text seems to describe data collection. It would read more sequentially if this section is renamed ‘data collection’ and is placed after the ‘patient and public involvement’ section.
  I understand the concern. The paragraph study design has been split in parts, belonging to the study design, study participants, and data collection and analysis.
- Study design: The method used is described as ‘a qualitative research method,’ this section would be strengthened by describing and referencing exactly what approach and methods were used and an explanation as to why they were chosen.
  This is further explained in the methods text within word count limits.
- Study design: A short statement justifying why the Decisional Needs Assessment questionnaire as a basis for the qualitative interview would be helpful and how this is in line with your chosen qualitative methods/approach.
  Idem.
- Study design: When describing the interviewers, it would be helpful to know of any characteristics which may influence their data collection and/or interpretation (i.e. researcher reflexivity).
  As the interviews are semi-structured and agreed upon by more than one reviewer, this will have limited effect on the results.
- Patient and public involvement: This heading is somewhat misleading as the text appears to describe the study sample and setting, not patient and public involvement. Re-naming the section ‘Study sample and setting’ would be clearer.
  The patient and public involvement statement has been changed and interchanges between different paragraphs of the methods have been made.
- Patient and public involvement: It would be interesting to know why convenience sampling was used and how many patients were excluded from the final sample.
  Convenience sampling was used, simply, as it was convenient. To the best of my knowledge, no exclusions were made. Obtainment of this information is limited due to recruitment through a third party.
- Data analysis: this section suggests that both thematic analysis and grounded theory approaches or methods were used. It would benefit from further explanation of how/if these two approaches/methods were integrated?
  These two methods were not integrated. Previously it was wrongly described.
- Data analysis: this section reports that the study is of an ‘explorative nature’, and together with the reference to grounded theory above, suggests that the aim is to derive meaning from the data. However the aim and findings appear more focussed and

descriptive. The section would benefit from a more comprehensive and consistent description of the data collection and analysis approach.
Idem.

- Data analysis: It would strengthen this section if there was a little more description of the process of open, axial and selective coding and how these were built into the themes described in the results section.
Idem.
- General characteristics: It would be useful to explain the relevance of reporting the number of patients/physicians who mentioned each theme/subtheme (including table 2), this seems a little unusual in a qualitative study which usually focusses on the meaning of themes rather than quantifying them.
It shows how prevalent a theme was as a quantifying measure of importance of that theme. It also shows some discrepancy between patients and physicians in what themes were important for each group.
- Themes: It would be interesting to know why numbers of participants relating to each theme/subtheme are reported, this seems a little unusual: see above comment
Idem.
- Themes: themes are clearly described however there does not appear to be an obvious analysis of meaning within the data or the development of a theory, which does not seem entirely consistent with the use of grounded theory.
See above comment.
- Themes, table 4: this is a helpful summary. Some of the quotations provide context to, and suggest subtleties in, decision making which are not entirely reflected in the results section, such as the influence on decision making of patient-physician relationships and patients' fears and anxieties. Further analysis of the qualitative data may result in a more in-depth understanding of decision making in CML.
This is an important discussion that should be further investigated. However, due to word count restraints and scope, this is out of scope of this paper. This is a great topic that could be revisited in future research.
- This section confirms that several findings are supported by wider literature. It would be interesting and relevant to also relate findings to literature specific to CML and haematological malignancies regarding patient/physician decision making/consultation, such as the mismatch of patient and physician perspectives on symptom burden in CML and unmet information needs in patients with haematological malignancies. Additionally, relating the findings to other qualitative work in CML and/or haematological malignancies would be particularly relevant.
To the best of my knowledge, I am not aware that such literature exists. I am very open to literature suggestions. Maybe this will give possibility for a deeper search of the literature in the future.
- This section would benefit from including more related literature related to CML and haematological malignancies, including qualitative studies.
Idem.
- There is no reference for thematic analysis, which if used, would be required.

The reference that was used has been added.

VERSION 2 - REVIEW

**Reviewer**	**2**
**Name**	**Hewison, Ann**
**Affiliation**	**University of York, Department of Health Sciences**
**Date**	**12-Dec-2024**
**COI**	**None**

Reviewer: 2
Dr. Ann Hewison, University of York
Comments to the Author:
Thank you for considering my previous comments on this paper. I am feel that most have been addressed adequately, however I have some remaining concerns (listed as 'UPDATED COMMENT' below, with the original comment and response) which I would consider minor corrections:
• Study design: When describing the interviewers, it would be helpful to know of any characteristics which may influence their data collection and/or interpretation (i.e. researcher reflexivity).
As the interviews are semi-structured and agreed upon by more than one reviewer, this will have limited effect on the results.
UPDATED COMMENT: I appreciate this view, however I believe the concept of reflexivity in qualitative research is important regardless of the number of researchers involved in data collection and analysis, as researcher biases and characteristics can still impact the research. A statement regarding this seems possible given there were three researchers in total, also this is a requirement of the SRQR reporting guidelines.
• Patient and public involvement: It would be interesting to know why convenience sampling was used and how many patients were excluded from the final sample.
Convenience sampling was used, simply, as it was convenient. To the best of my knowledge, no exclusions were made. Obtainment of this information is limited due to recruitment through a third party.
UPDATED COMMENT: (This section is now renamed 'Study participants'). If none of those patients or physicians contacted by the research team were excluded at this point, then a statement confirming this would support this section.

The new section titled ‘Patient and public involvement’ may not have fully understood the definition of this term. As I understand it, this refers to Patient and public involvement members who contribute to the design, implementation and dissemination of the study and are not research participants (see: https://www.learningforinvolvement.org.uk/content/resource/a-brief-guide-to-patient-and-public-involvement-and-qualitative-methods-in-health-and-social-care-research/)

• Data analysis: this section reports that the study is of an ‘explorative nature’, and together with the reference to grounded theory above, suggests that the aim is to derive meaning from the data. However the aim and findings appear more focussed and descriptive. The section would benefit from a more comprehensive and consistent description of the data collection and analysis approach.

Idem.

UPDATED COMMENT: It is helpful that the analytical approach is now defined and I appreciate that there is a limit to this section in terms of word count. However, some detail on the approach to thematic analysis would support this section. For example, was complete coding carried out? was data derived and/or researcher derived coding used? was the process iterative? how were themes developed – e.g. comparison of codes, looking for patterns in the data etc.?

• General characteristics: It would be useful to explain the relevance of reporting the number of patients/physicians who mentioned each theme/subtheme (including table 2), this seems a little unusual in a qualitative study which usually focusses on the meaning of themes rather than quantifying them.

It shows how prevalent a theme was as a quantifying measure of importance of that theme. It also shows some discrepancy between patients and physicians in what themes were important for each group.

UPDATED COMMENT: I agree that an idea of the proportion of participants relating to each theme is of interest to readers however, as I understand it, the importance of a theme in qualitative research is how well it can explore and relate to the research question rather than just the number of participants it relates to (Nowell et al 2017:https://doi.org/10.1177/1609406917733847). Qualitative methods cannot be used to infer statistical generalisability by suggesting the proportion of participants relating to a qualitative theme would be the same in the general population, quantitative methods are better suited to this. The paper overall seems to over-emphasise the frequency of participants per subtheme.

• Themes: It would be interesting to know why numbers of participants relating to each theme/subtheme are reported, this seems a little unusual: see above comment

Idem.

UPDATED COMMENT: See my previous comment

• Themes, table 4: this is a helpful summary. Some of the quotations provide context to, and suggest subtleties in, decision making which are not entirely reflected in the results section, such as the influence on decision making of patient-physician relationships

and patients' fears and anxieties. Further analysis of the qualitative data may result in a more in-depth understanding of decision making in CML.
This is an important discussion that should be further investigated. However, due to word count restraints and scope, this is out of scope of this paper. This is a great topic that could be revisited in future research.
UPDATED COMMENT: I understand this point. An explanation about this may help the reader understand why a more in-depth analysis was not carried out.
• Discussion - This section confirms that several findings are supported by wider literature. It would be interesting and relevant to also relate findings to literature specific to CML and haematological malignancies regarding patient/physician decision making/consultation, such as the mismatch of patient and physician perspectives on symptom burden in CML and unmet information needs in patients with haematological malignancies. Additionally, relating the findings to other qualitative work in CML and/or haematological malignancies would be particularly relevant.
To the best of my knowledge, I am not aware that such literature exists. I am very open to literature suggestions. Maybe this will give possibility for a deeper search of the literature in the future.
UPDATED COMMENT: I am aware of literature relating to the topics mentioned. I believe it should be relatively straightforward to find some relevant papers using a standard database. These are some of several examples:
Efficace, F. et al. (2014). Patient- versus physician-reporting of symptoms and health status in chronic myeloid leukemia. Haematologica, 99 (4), pp.788–793
Graffigna, G. et al. (2017). Recovering from chronic myeloid leukemia: the patients' perspective seen through the lens of narrative medicine. Quality of Life Research: An International Journal of Quality of Life Aspects of Treatment, Care and Rehabilitation, 26 (10), pp.2739–2754.
Hall, A. et al. (2013). Supportive care needs of hematological cancer survivors: a critical review of the literature. Critical Reviews in Oncology/Hematology, 88 (1), pp.102–116.

• This section would benefit from including more related literature related to CML and haematological malignancies, including qualitative studies.
Idem.
UPDATED COMMENT: Please see my previous comment

Reviewer: 2
If you have selected 'Yes' above, please provide details of any competing interests.: Not applicable

---

VERSION 2 - AUTHOR RESPONSE

Our response to how we took the comments on this paper in consideration is described in *cursive*. The first bullet point was the comment made in the first round, the open bullet the response given by the now second-author, and the updated comment was feedback provided in the second round.

Reviewer: 2
Dr. Ann Hewison, University of York
Comments to the Author:
Thank you for considering my previous comments on this paper. I am feel that most have been addressed adequately, however I have some remaining concerns (listed as 'UPDATED COMMENT' below, with the original comment and response) which I would consider minor corrections:

- Study design: When describing the interviewers, it would be helpful to know of any characteristics which may influence their data collection and/or interpretation (i.e. researcher reflexivity).
    - As the interviews are semi-structured and agreed upon by more than one reviewer, this will have limited effect on the results.
        - UPDATED COMMENT: I appreciate this view, however I believe the concept of reflexivity in qualitative research is important regardless of the number of researchers involved in data collection and analysis, as researcher biases and characteristics can still impact the research. A statement regarding this seems possible given there were three researchers in total, also this is a requirement of the SRQR reporting guidelines.

We added a paragraph under "researcher characteristics and reflexivity", in which we described the researchers, our research focus and context, which might have oriented questioning and interpretation in those directions our group was most familiar with.

- Patient and public involvement: It would be interesting to know why convenience sampling was used and how many patients were excluded from the final sample.
    - Convenience sampling was used, simply, as it was convenient. To the best of my knowledge, no exclusions were made. Obtainment of this information is limited due to recruitment through a third party.
        - UPDATED COMMENT: (This section is now renamed 'Study participants'). If none of those patients or physicians contacted by the research team were excluded at this point, then a statement confirming this would support this section.
          The new section titled 'Patient and public involvement' may not have fully understood the definition of this term. As I understand it, this refers to Patient and public involvement members who

contribute to the design, implementation and dissemination of the study and are not research participants (see: https://www.learningforinvolvement.org.uk/content/resource/a-brief-guide-to-patient-and-public-involvement-and-qualitative-methods-in-health-and-social-care-research/)

In the section "patient and public involvement", the role of the patients, especially those at the base of this research initiative, are described. Besides, in the "general characteristics" section of the results, we described that none of the recruited patients were excluded.

- Data analysis: this section reports that the study is of an 'explorative nature', and together with the reference to grounded theory above, suggests that the aim is to derive meaning from the data. However the aim and findings appear more focussed and descriptive. The section would benefit from a more comprehensive and consistent description of the data collection and analysis approach.
    - Idem.
        - UPDATED COMMENT: It is helpful that the analytical approach is now defined and I appreciate that there is a limit to this section in terms of word count. However, some detail on the approach to thematic analysis would support this section. For example, was complete coding carried out? was data derived and/or researcher derived coding used? was the process iterative? how were themes developed – e.g. comparison of codes, looking for patterns in the data etc.?

Under the section "data collection and analysis" the analysis process is described in more detail, describing that we used inductive coding on selections of the transcripts relevant to the aim of the research. The analysis was performed following the codebook thematic analysis technique, with the deviation of inductive coding. The formation of themes is described as well, which was done through comparing codes within and across transcripts, across coders, which were after clustered based on their relations and patterns.

- General characteristics: It would be useful to explain the relevance of reporting the number of patients/physicians who mentioned each theme/subtheme (including table 2), this seems a little unusual in a qualitative study which usually focusses on the meaning of themes rather than quantifying them.
    - It shows how prevalent a theme was as a quantifying measure of importance of that theme. It also shows some discrepancy between patients and physicians in what themes were important for each group.
        - UPDATED COMMENT: I agree that an idea of the proportion of participants relating to each theme is of interest to readers

however, as I understand it, the importance of a theme in qualitative research is how well it can explore and relate to the research question rather than just the number of participants it relates to (Nowell et al 2017:https://doi.org/10.1177/1609406917733847). Qualitative methods cannot be used to infer statistical generalisability by suggesting the proportion of participants relating to a qualitative theme would be the same in the general population, quantitative methods are better suited to this. The paper overall seems to over-emphasise the frequency of participants per subtheme.

We revisited and rediscussed our themes and subthemes. The smaller subtheme "personal experiences", was reflected upon and divided among other similar themes. Besides, the numbering was removed and more descriptive words were used to describe the weight it carried for the group of respondents, and to still show the differences between HCP and patients in how they viewed the (sub)themes

- Themes: It would be interesting to know why numbers of participants relating to each theme/subtheme are reported, this seems a little unusual: see above comment
    - Idem.
        - UPDATED COMMENT: See my previous comment

See my previous answer

- Themes, table 4: this is a helpful summary. Some of the quotations provide context to, and suggest subtleties in, decision making which are not entirely reflected in the results section, such as the influence on decision making of patient-physician relationships and patients' fears and anxieties. Further analysis of the qualitative data may result in a more in-depth understanding of decision making in CML.
    - This is an important discussion that should be further investigated. However, due to word count restraints and scope, this is out of scope of this paper. This is a great topic that could be revisited in future research.
        - UPDATED COMMENT: I understand this point. An explanation about this may help the reader understand why a more in-depth analysis was not carried out.

The conclusion is now written more in line with the goal of this research aim. It described the focus of this research on providing recommendations for a decision-aid specifically. It also recommends to examine whether these recommendations can improve patient-clinician communication and whether CML patients feel empowered in the SDM process.

- Discussion - This section confirms that several findings are supported by wider literature. It would be interesting and relevant to also relate findings to literature specific to CML and haematological malignancies regarding patient/physician decision making/consultation, such as the mismatch of patient and physician perspectives on symptom burden in CML and unmet information needs in patients with haematological malignancies. Additionally, relating the findings to other qualitative work in CML and/or haematological malignancies would be particularly relevant.
    - To the best of my knowledge, I am not aware that such literature exists. I am very open to literature suggestions. Maybe this will give possibility for a deeper search of the literature in the future.
        - UPDATED COMMENT: I am aware of literature relating to the topics mentioned. I believe it should be relatively straightforward to find some relevant papers using a standard database. These are some of several examples:
          Efficace, F. et al. (2014). Patient- versus physician-reporting of symptoms and health status in chronic myeloid leukemia. Haematologica, 99 (4), pp.788–793
          Graffigna, G. et al. (2017). Recovering from chronic myeloid leukemia: the patients' perspective seen through the lens of narrative medicine. Quality of Life Research: An International Journal of Quality of Life Aspects of Treatment, Care and Rehabilitation, 26 (10), pp.2739–2754.
          Hall, A. et al. (2013). Supportive care needs of hematological cancer survivors: a critical review of the literature. Critical Reviews in Oncology/Hematology, 88 (1), pp.102–116.

I sincerely thank you for your literature suggestions. I read these, and saw a lot of overlaps, or additions to our own research topic. We included references to some of these suggestions in our final article and described how our research relates or contributes to existing research fields.

- This section would benefit from including more related literature related to CML and haematological malignancies, including qualitative studies.
    - Idem.
        - UPDATED COMMENT: Please see my previous comment

See comment above
